# Plant Responses to Heat Stress: Physiology, Transcription, Noncoding RNAs, and Epigenetics

**DOI:** 10.3390/ijms22010117

**Published:** 2020-12-24

**Authors:** Jianguo Zhao, Zhaogeng Lu, Li Wang, Biao Jin

**Affiliations:** 1 College of Horticulture and Plant Protection, Yangzhou University, Yangzhou 225009, China; zhaojg@yzu.edu.cn (J.Z.); d160068@yzu.edu.cn (Z.L.); liwang@yzu.edu.cn (L.W.); 2 Guangling College of Yangzhou University, Yangzhou University, Yangzhou 225009, China

**Keywords:** heat stress, physiological, molecular, non-coding RNA, epigenetics

## Abstract

Global warming has increased the frequency of extreme high temperature events. High temperature is a major abiotic stress that limits the growth and production of plants. Therefore, the plant response to heat stress (HS) has been a focus of research. However, the plant response to HS involves complex physiological traits and molecular or gene networks that are not fully understood. Here, we review recent progress in the physiological (photosynthesis, cell membrane thermostability, oxidative damage, and others), transcriptional, and post-transcriptional (noncoding RNAs) regulation of the plant response to HS. We also summarize advances in understanding of the epigenetic regulation (DNA methylation, histone modification, and chromatin remodeling) and epigenetic memory underlying plant–heat interactions. Finally, we discuss the challenges and opportunities of future research in the plant response to HS.

## 1. Introduction

Plants as sessile organisms cannot move to favorable environments upon encountering abiotic or biotic stresses; consequently, plant growth, development, and productivity are markedly affected [1]. High temperature is an important stress and global warming has accelerated the increase in air temperature in recent decades [2]. Therefore, the mechanisms by which plants respond to high temperature are of great interest. Plants exposed to high temperature (heat stress, HS) suffer from severe, and sometimes lethal, adverse effects. To cope with such conditions, plants have evolved sophisticated mechanisms to respond to HS. For example, several basic physiological processes of plants—including photosynthesis, respiration, and water metabolism—respond to HS [3,4]. Much progress has been made in characterizing HS-responsive genes, non-coding RNAs, DNA methylation, and histone modifications. Here, we focus on the physiological, transcriptional, post-transcriptional, and epigenetic mechanisms underlying the plant HS response.

## 2. Physiological Responses of Plants under HS

**Figure 1:**
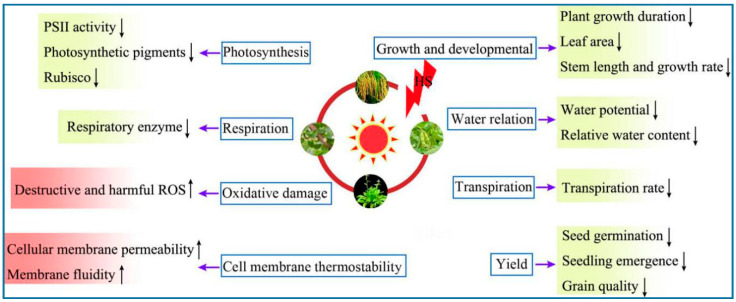
Effects of heat stress on plant physiological responses. Upward-pointing arrows indicate activated/upregulated physiological indices. Downward-pointing arrows indicate deactivated/downregulated physiological indices. Abbreviations: HS, heat stress; PSII, photosystem II; Rubisco, ribulose-1,5-bisphosphate carboxylase/oxygenase; ROS, reactive oxygen species.

A variety of physiological processes—such as photosynthesis, respiration, transpiration, membrane thermostability, and osmotic regulation—are adversely affected by HS. Some common effects of HS on plant physiological responses, growth and development, and yield are shown in Figure 1.

### 2.1. Photosynthesis

Generally, HS reduces photosynthetic efficiency, thus shortening the plant life cycle and diminishing productivity [5]. Photosynthesis is also one of the most heat-sensitive physiological processes. Under HS, photochemical reactions in thylakoid lamellae and carbon metabolism in the stroma of chloroplasts are prone to injury [6,7]. Heat stress causes disruption of thylakoid membranes, thereby inhibiting the activities of membrane-associated electron carriers and enzymes, reducing the rate of photosynthesis. Specifically, photosystem II (PSII) activity is greatly reduced or even stops under HS because PSII complex is the most heat-intolerant [6,8]. In addition, HS influences chloroplast structure and the thermal stability of components of the photosynthetic system, reducing ribulose-1,5-bisphosphate carboxylase/oxygenase (Rubisco) activity, amounts of photosynthetic pigments, and the carbon fixation capacity [6,9,10]. These factors contribute significantly to the reduction of photosynthetic efficiency under HS. Therefore, a fundamental understanding of the response of photosynthetic physiology is helpful to study the thermostability of plants and the adverse effects of warming on crop yield [11].

### 2.2. Cell Membrane Thermostability

Membrane dysfunction is the main physiological consequence of plant exposure to HS. Under extreme HS, the increased kinetic energy and movement of biomolecules across membranes loosens chemical bonds, leading to disintegration of membrane lipids and increasing membrane fluidity [12]. HS increases cellular membrane permeability and the loss of cellular electrolytes, consequently inhibiting cellular function and decreasing thermotolerance [5]. In addition, the reactive oxygen species (ROS) accumulation caused by HS leads to membrane damage, decreasing thermotolerance [13]. In short, membrane thermostability plays an important role in conferring tolerance to HS in plants.

### 2.3. Oxidative Damage

Plants exposed to HS show accumulation of ROS—singlet oxygen (^1^O_2_), superoxide radical (O_2_^−^), hydrogen peroxide (H_2_O_2_), and hydroxyl radical (OH^−^)—generating oxidative stress [14]. The ROS are generated mainly in PSI and PSII. In PSII, excess energy generates the triplet state of chlorophylls, which pass excitation energy to O_2_, producing singlet oxygen. Over-reduction of PSI leads to generation of the superoxide anion, promoting H_2_O_2_ production [8]. ROS (e.g., O_2_^−^, H_2_O_2_) induce oxidative stress by altering membrane properties, degrading proteins, and inactivating enzymes, thus reducing plant cell viability [15]. Heat stress induces lipid peroxidation due to free radical damage of the cell membrane [6]. Under HS, the content of malondialdehyde (MAD; an indicator of lipid peroxidation) is significantly increased in many plants such as sorghum [16]. ROS can also trigger programmed cell death under HS. On the other hand, plants have developed mechanisms to detoxify ROS and enhance heat tolerance. Plants increase their thermotolerance by recruiting the antioxidant enzymes superoxide dismutase (SOD), ascorbate peroxidase (APX), catalase (CAT), glutathione reductase (GR), and peroxidase (POX) [17].

### 2.4. Other Physiological Responses

Plant water status is generally erratic under changing temperatures [5]. Heat stress causes dehydration and affects plant growth and development. Water potential and relative water content are substantially decreased upon exposure to HS, reducing photosynthetic productivity [3]. However, under transient or mild HS, plants regulate the rate of respiration and transpiration to balance water loss and heat dissipation. The level of soluble sugars and proteins are also altered during HS to regulate osmotic pressure within the cell [18]. Finally, HS reduces the yield of cultivated crops, including cereals, legumes, and oil crops [19].

## 3. Molecular Responses of Plants under HS

### 3.1. Transcriptional Regulation of HS Responses

When plants are subjected to HS, the expression of a series of heat shock transcription factor (HSF) and heat shock protein (HSP) genes is induced. The *HSFs* rapidly induce the expression of *HSPs*, and both *HSFs* and *HSPs* play central roles in the plant HS response and induction of thermotolerance [20,21]. However, overexpression of a single *HSF* or *HSP* gene has little impact on thermotolerance, suggesting that *HSFs* and *HSPs* act synergistically to confer HS resistance.

**Figure 2:**
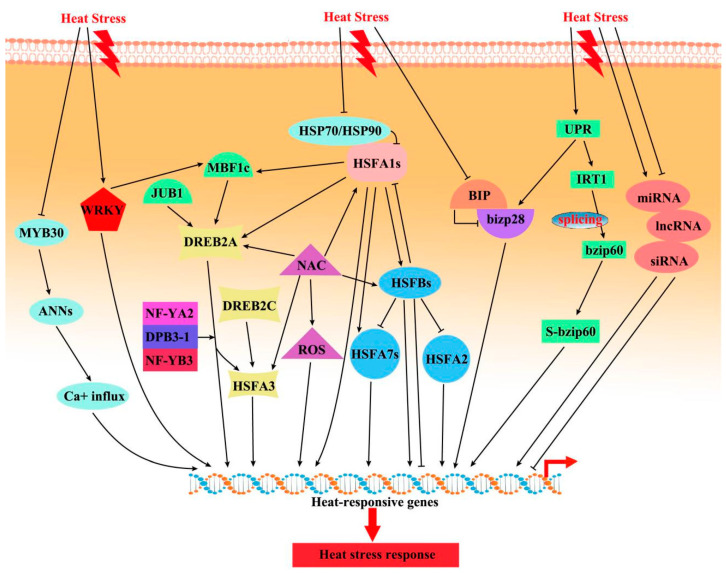
Schematic of the main regulatory pathways that respond to HS transcription factors. The binding of heat shock protein (HSP)70/90 and heat shock transcription factor (HSF)A1s represses the activity of HSFA1s under nonstress conditions, whereas heat stress elicits the dissociation of HSFA1 from HSP70 and HSP90, leading to HSFA1 activation. Abbreviations: HSP, heat shock protein; HSF, heat shock transcription factor; ANN, annexin; JUB1, jungbrunnen 1; MBF1c, multiprotein-bridging factor 1c; DREB2A/2C dehydration-responsive element binding protein 2A/2C; NF-Y, nuclear factor Y; DPB3-1, DNA polymerase II subunit B3-1; ROS, reactive oxygen species; BIP, binding immunoglobulin protein; bIZP, basic leucine zipper; S-bzip60, spliced bZIP60; UPR, unfolded protein response; IRT1, inositol-requiring enzyme 1; miRNA, microRNA; lncRNA, long non-coding RNA; siRNA, small interfering RNA.

Plant HSFs are divided into three conserved evolutionary classes (A, B, and C) according to the structural features of their oligomerization domains. Class A HSFs are essential for transcriptional activation. However, Class B and C HSFs have no activator function because they lack the appropriate motif comprising acidic amino acid residues [22]. Among class A HSFs, HSFA1 is the master transcriptional activator, triggering the immediate expression of other HS-responsive transcription factors (TFs) [20], including DEHYDRATION-RESPONSIVE ELEMENT BINDING PROTEIN 2A (DREB2A), HSFA2, HSFA7, HSFBs, and MULTIPROTEIN-BRIDGING FACTOR 1C (MBF1C) (Figure 2). HSFA1 transactivation activity is induced by interaction with HEAT SHOCK PROTEIN 70 (HSP70) and HSP90 under HS [23]. Interestingly, both HSFA1a and HSFA1b are important for the initial phase of HS-responsive gene expression [24]. HSFA2, as a heat-inducible transactivator, prolongs acquired thermotolerance by maintaining the expression of *HSP* genes in *Arabidopsis* [25]. HSFA3 is regulated by DREB2A and DREB2C, playing a role in thermotolerance [20,26]. DREB2A, a key transcription factor, directly regulates HSFA3 transcription via a coactivator complex of NUCLEAR FACTOR Y, SUBUNIT A2 (NF-YA2), NF-YB3, and DNA POLYMERASE II SUBUNIT B3-1 (DPB3-1)/NF-YC10 under HS (Figure 2). In addition, HSFA4a and HSFA8 act as sensors of the ROS produced as secondary stress responses during the HS response in *Arabidopsis* [27].

Among class B HSFs, HSFBs are transcriptional repressors and negatively regulate the expression of many heat-inducible *HSFs* (*HSFA2*, *HSFA7s*) and *HSPs* (e.g., *HSP101*, *HSP70*). In addition, HSFBs are downstream target genes of HSFA1s in plants, and they influence and interact with each other, forming the regulatory network responsible for the expression of HS-responsive genes (Figure 2), for instance, in *Arabidopsis*, tomato, and tall fescue (*Festuca arundinacea*) [23,28,29]. The functions and roles of class C HSFs are unclear. In wheat, overexpression of *TaHSFC2a-B* resulted in upregulation of *HSPs* and other heat protection genes (e.g., *TaHSP70d* and *TaGalSyn*) and improved thermotolerance. Overexpression of *FaHSFC1b* (cloned from tall fescue) in *Arabidopsis* enhanced heat tolerance by inducing or upregulating the expression of *HSPs* [29]. In addition, HSFC genes are upregulated by HS in wheat [30], cabbage (*Brassica rapa*) [31], and soybean (*Glycine max*) [32].

Other TF families, such as MBF1C, NAC, WRKY, bZIP (basic leucine zipper), and MYB, are also involved in the regulation of heat-responsive genes (Figure 2). MBF1C is a highly conserved transcriptional coactivator and a key regulator of thermotolerance [33]. Indeed, an *mbf1c* mutant had reduced the expression levels of DREB2A and HSFBs during HS. In addition, HSFA1s regulate the expression of MBF1C during HS. NACs are one of the largest transcription factor families in plants and are involved in the response to HS. NAC transcription factors bind to the promoters of *HSFs* (e.g., *HSFA1b*, *HSFA6b*, *HSFA7a*, and *HSFC1*), increasing their expression and thus enhancing thermotolerance [34,35]. Moreover, *TaNAC2L* enhanced heat resistance by regulating the expression of HS-response genes (e.g., *AtHSFA3*, *AtDREB2A*) in wheat [36] (Figure 2). Interestingly, the overexpression of *OsNAC3* in rice enhanced tolerance to HS by modulating ROS homeostasis [37]. The NAC transcription factor JUNGBRUNNEN1 (JUB1) regulates the expression of DREB2A under HS [38].

Notably, a minimal yet significant level of acquired thermotolerance can be attained in plants by induction of the expression of a small number of genes regulated by other transcription factors such as WRKY, bZIP, and MYB. WRKYs participate in developmental and physiological processes, as well as in stress responses. Under HS, WRKY18, WRKY25, WRKY26, WRKY33, WRKY39, WRKY40, WRKY46, and WRKY68 coordinately induce plant thermotolerance by positively regulating HSP-related signaling pathways (e.g., HSFs, HSPs, and MBF1c) [39,40]. In addition, OsWRKY11 in rice plays a role in the HS response and tolerance. Overexpression of OsWRKY11 under the control of the HSP101 promoter led to enhanced heat tolerance [41].

The bZIP transcription factors and the unfolded protein response (UPR) play important roles in plant thermotolerance. bZIP28 and bZIP60, which localize to the endoplasmic reticulum (ER) membrane, are transferred to the nucleus, where they activate the expression of stress-responsive genes [42]. Under HS, the ER membrane-localized RNA splicing factor IRE1 (INOSITOL-REQUIRING ENZYME 1) splices the mRNA of *bZIP60*, causing synthesis of a spliced bZIP60 (sbZIP60), which translocates into the nucleus [28]. The ER-localized chaperone BiP (BINDING PROTEIN) binds to bZIP28 and inhibits its activation under non-stress conditions. The coordination of bZIP28 and HSFA2 is involved in regulation of the HS response in *Arabidopsis*. bZIP28-deficient plants showed enhanced activation of cytosolic APX-, MBF1c-, HSP-dependent pathways, and had elevated HSFA2 transcript levels, suggesting these pathways compensate for the deficiency in bZIP28 during HS [43]. The activation of bZIP17 is controlled by HS in a manner similar to the regulatory mechanism that controls the UPR. In lily (*Lilium longiflorum*), promotion of thermotolerance by LlHSFAs involves regulation of bZip factors (AtbZIP11, AtbZIP44) [44]. In addition, the response pathway of bZIPs is activated during prolonged warming [19].

MYBs are involved in plant development, metabolism, and stress responses. MYB30 regulates HS responses via ANNEXIN (ANN)-mediated cytosolic calcium signaling in *Arabidopsis* [45]. MYB30 binds to the promoters of *ANN1* and *ANN4* and represses their expression. Subsequently, ANNs modulate heat-induced [Ca^2+^]_cyt_ elevation, triggering downstream HS responses (Figure 2). In addition, the overexpression in rice of OsMYB55 increased the accumulation of amino acids (e.g., glutamic acid, gamma aminobutyric acid, arginine, and proline), further improving the heat resistance of rice at the vegetative stage [46].

### 3.2. Regulation of HS Responses by Non-Coding RNAs

**Figure 3:**
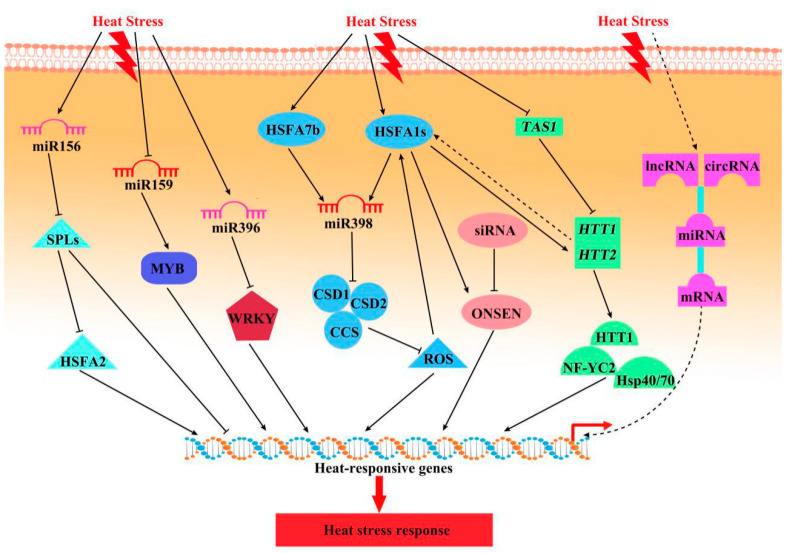
Roles of non-coding RNAs in the plant HS response. Dotted lines represent as yet unidentified factors in the corresponding pathways. Abbreviations: SPL, squamosa promoter-binding protein-like; HSF, heat shock transcription factor; CSD, copper/zinc superoxide dismutase; CCS, copper chaperone for superoxide dismutase; ROS, reactive oxygen species; TAS1, trans-acting siRNA precursor 1; HTT, heat-induced tas1 target; NF-YC2, nuclear factor Y, subunit C; lncRNA, long non-coding RNA; circRNA, circular RNA; siRNA, small interfering RNA; miRNA, microRNA; mRNA, messenger RNA.

Although transcription factors are the core regulators of transcription during HS, plant non-coding RNAs (ncRNAs) play an important role in the response to HS (Figure 3). ncRNAs are a class of regulatory RNAs comprising microRNAs (miRNAs), small interfering RNAs (siRNAs), long non-coding RNAs (lncRNAs), and circular RNAs (circRNAs) [47]. ncRNAs play important roles in the HS response by regulating the activity of TF s or genes.

MicroRNAs are a class of small ncRNAs that negatively regulate gene expression by degrading mRNA or inhibiting translation [48]. miR156 isoforms are induced by HS and are important for HS memory. During recovery from HS, miR156 sustains the expression of HS-responsive genes (e.g., *HSFA2* and *HSPs*) via *SPL*s in *Arabidopsis* [49]. As a result of conservation of miR156, the miR156-SPL module that regulates HS memory is conserved in plants. By contrast, miR398 is rapidly induced in response to HS, downregulating its target genes (*CSD1/2*, copper/zinc superoxide dismutase1/2; *CCS*, copper chaperone for superoxide dismutase) [50]. This leads to ROS accumulation and increased HSF and HSP levels. Given that miR398 expression is under the control of HSFAs, this regulatory mechanism constitutes a positive feedback loop. In addition, miR398 and its target *CSDs* are implicated in the HS responses of *Brassica rapa* and *Populus tomentosa* [51]. miR159 is rapidly induced in wheat exposed to HS [52]. GAMYB TFs, the main targets of miR159, have roles in heat tolerance. Indeed, transgenic wheat overexpressing miR159 and a *myb33myb65 Arabidopsis* mutant showed increased susceptibility to HS, indicating that miR159 regulates plant heat tolerance by *GAMYB* targets. miR396 regulates HaWRKY6 during early responses to HS, and transgenic *Helianthus annuus* plants expressing a miR396-resistant HaWRKY6 gene exhibited enhanced tolerance to HS [53].

Small interfering RNAs, approximately 21–24-nucleotide endogenous RNAs, are involved in plant responses to abiotic stresses. They are classified into *trans*-acting small interfering RNAs (ta-siRNAs, TAS), natural antisense transcript siRNAs (nat-siRNAs), and heterochromatic siRNAs [51,54]. In *Arabidopsis*, the *copia-like* retrotransposon *ONSEN* was activated in siRNA biogenesis mutants during HS, likely because *ONSEN* is a target of HSFA1 and HSFA2. In addition, new *ONSEN* insertions were detected in progeny after HS, and conferred heat-responsiveness to genes near the new insertion sites, suggesting a role for *ONSEN* transposition in transgenerational HS memory [55]. ta-siRNAs are distinctive siRNAs generated by miRNA processing of a noncoding *TAS* precursor RNA. HEAT-INDUCED TAS1 TARGET1 (HTT1) and HTT2 are involved in thermotolerance and are targeted by TAS1 (trans-acting siRNA precursor 1)-derived siRNAs (Figure 3). Under HS, TAS1 negatively regulates *HTT1* and *HTT2* and reduces thermotolerance. In addition, HSFA1a also directly activates *HTT1* and *HTT2* by binding to their promoters, inducing thermotolerance [56]. In *Brassica rapa*, nat-siRNAs derived from *cis*-NATs were responsive to HS, suggesting that nat-siRNAs may play important roles in heat resistance [57].

lncRNAs and circRNAs function as competitive endogenous RNAs and are regulated by competition for binding to common miRNA response elements [58]. lncRNAs are diverse transcripts longer than 200 nt and are vital in the plant HS response. The expression level of the lncRNA PsiLncRNA00268512 was dynamic in response to HS in *P*. *simonii* [59]. In addition, in *B*. *rapa*, competition between lncRNAs and protein-coding genes for binding to miR159a or miR172a regulates target genes or heat-responsive genes (e.g., HSPs, HSFs, and DREB2A) [60]. circRNAs are single-stranded RNAs in closed circular form and are involved in regulation of the plant response to HS. In *Cucumis sativus*, many miRNAs, with mRNAs, lncRNAs, and circRNAs, are associated with plant hormone signal transduction pathways in response to HS [58]. However, the lncRNA and circRNA responses to HS are unclear, particularly the lncRNA/circRNA–miRNA–mRNA coexpression network (Figure 3).

### 3.3. Epigenetic Regulation in the Plant HS Response and Memory

**Figure 4:**
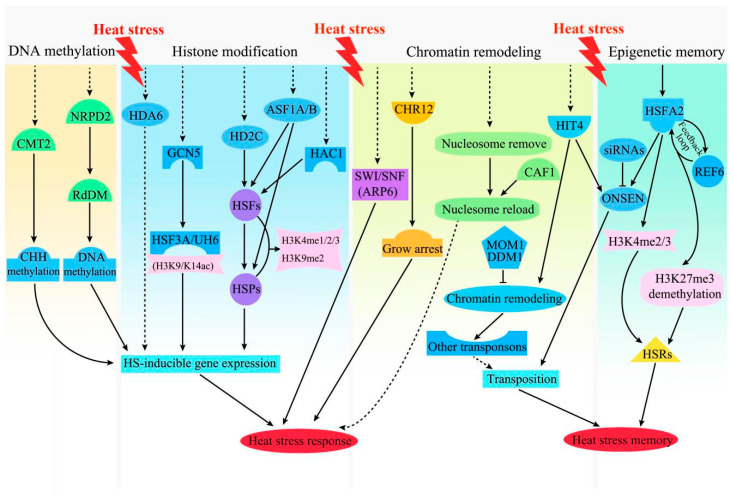
Epigenetic regulation of the plant response to HS, including DNA methylation, histone modification, chromatin remodeling, and epigenetic memory. Dotted lines represent as yet unidentified factors in the corresponding pathways. Abbreviations: CMT2, chromomethylase 2; NRPD2, nuclear RNA polymerase D2; RdDM, RNA-directed DNA methylation; HDA6, histone deacetylase 6; GCN5, general control nonderepressible 5; HD2C, histone deacetylase 2C; ASF1A/B, anti-silencing function 1A/B; HAC1, histone acetyltransferase 1; SWI/SNF, SWItch/sucrose non-fermentable; ARP6, actin-related protein 6; CHR, chromatin remodeling; CAF1, chromatin assembly factor 1; MOM1, morpheus’ molecule 1; DDM1, decrease in DNA methylation 1; HIT4, heat-intolerant 4; REF6, relative of early flowering 6; H3K4/9/27me, H3K4/9/27 methylation; H3K9/K14ac, H3K9/K14 acetylation; HSR, heat stress response.

Epigenetics refers to the heritable changes in gene expression that occur without DNA sequence variations and are pivotal for the plant HS response [61,62]. The epigenetic regulatory system in response to heat involves DNA methylation, histone modification, chromatin remodeling (Figure 4), sRNAs, and lncRNAs, which alter the gene expression pattern and/or epigenetic memory of plants under HS [63,64].

#### 3.3.1. DNA Methylation

DNA methylation involves the transfer of a methyl group (CH_3_) to the cytosine position of DNA to form 5-methylcytosine, forming CG, CHG, and CHH (H represents A, T, or C) [65]. DNA methylation is involved in the regulation of genes implicated in the plant response to HS [63,66,67]. Upon exposure to HS, genome-wide methylation is increased significantly in *Arabidopsis thaliana* and *Quercus suber* under extreme heat [68,69]. In *Brassica napus*, the levels of DNA methylation increased more in a heat-sensitive than a heat-tolerant genotype under HS [70]. By contrast, decreased expression of S-ADENOSYL-L-HOMOCYSTEINE HYDROLASE1 (SAHH1) and DNA methyltransferases under HS reduced genome-wide DNA methylation in a heat-sensitive compared to a heat-tolerant (HT) cotton line [71]. Comparison of the methylation of cotton anthers at different stages in heat-sensitive and HT lines suggests that global disruption of DNA methylation (especially CHH methylation) in the heat-sensitive line at high temperatures leads to male sterility [72]. Interestingly, cold-acclimated *B*. *rapa* alters DNA methylation patterns to enhance its heat tolerance and maintain growth [67]. Therefore, global methylation is affected differently by heat among plant species and is linked to plant development.

Analysis of the heat tolerance of DNA methylation-deficient mutants revealed that the RNA-directed DNA methylation (RdDM) pathway is required for basal thermotolerance [66]. *Arabidopsis* plants deficient in *DRM1*/*DRM2* (domains rearranged methyltransferase 1/2) and *CMT3* (chromomethylase 3) are less sensitive to HS, but mutants in *nrpd2* (nuclear RNA polymerase D 2), *dcl3* (dicer-like 3), *rdr2 (*RNA-dependent RNA polymerase 2), and *ago4* (argonaute 4), which are involved in the RdDM pathway, are hypersensitive to heat (Figure 4). Further studies on *nrpd2* mutants recovering from heat revealed that the misexpression of heat-dependent genes is correlated with defective epigenetic regulation of adjacent transposon remnants. For instance, in *nrpd2* mutants, transcriptional activity of the COPIA-like transposon At1g29475 was increased by heat but did not decrease during recovery. By contrast, the expression of auxin-responsive genes near the transposon was downregulated during recovery of *nrpd2* plants [66]. Similarly, the heat-responsive LTR-copia type retrotransposon ONSEN, which is enhanced in several RdDM pathways, confers heat-responsiveness to genes close to the new insertion site [55]. Moreover, the expression of *Calmodulin-like 41* (*CML41*, *At3g50770*) is enhanced by HS and shows a reduced methylation level in the transposable element (TE) insertion close to the transcriptional start site [73]. Therefore, the RdDM pathway affects the transcription of genes near transposons or containing TEs by changing their DNA methylation status, which can improve plant basal thermotolerance. By contrast, plants deficient in *CMT2*, which is responsible for CHH methylation, have improved HS tolerance (Figure 4), suggesting that CMT2-dependent CHH methylation alleviates the plant response to HS [74].

#### 3.3.2. Histone Modification

Histone octamers comprise two copies of H2A, H2B, H3, and H4, wrapped in ≈147 bp DNA, forming the basic structural unit in nucleosome of chromatin. Histone acetylation and methylation mediate the plant HS response by repressing or activating gene expression. In *Chlamydomonas reinhardtii*, a high level of histone H3/4 acetylation (H3/4ac) and a low level of H3K4 methylation 1 (H3K4me1) were found in the promoter regions of active genes after HS [75]. By contrast, the level of acetylated histone H3 was decreased in the cork oak tree exposed to 45 °C. Similarly, the levels of H3K9me2 and H3K4me3 are significantly reduced after prolonged HS in *Arabidopsis* [76]. Moreover, the H3K9me2 level of *OsFIE1*, which is related to rice seed development, is temperature-sensitive (moderate HS, 34 °C) and may regulate *OsFIE1* expression for rice seed development [77]. Therefore, these two histone modifications are differently affected by heat among plant species.

Several epigenetic regulators—such as acetyltransferases, methyltransferases, deacetylases, and demethylases—mediate methylation and acetylation during the HS response [78]. These epigenetic regulators are recruited by heat response-associated recruiters (e.g., TFs, lncRNAs) to specific histones in chromatin to regulate gene expression [64,79]. *A*. *thaliana* plants deficient in acetyltransferase GCN5 exhibit serious defects in thermotolerance under HS, and *GCN5* may positively regulate thermotolerance by facilitating H3K9/K14 acetylation in the promoter regions of *HSFA3* and *ULTRAVIOLET HYPERSENSITIVE6* (Figure 4) [80]. Histone deacetylase 6 (HDA6), an RPD3-type deacetylase, is implicated in repression of gene expression by RdDM [81]. *hda6* mutants were hypersensitive to extreme heat, suggesting HDA6 is required for HS [82]. The transcript level of the histone deacetylase, HD2C, was increased in *Arabidopsis* subjected to heat treatment (Figure 4). An *hd2c* mutant analysis showed that HD2C downregulates heat-activated genes, implicating HD2C in regulation of the plant HS response. Because HD2C interacts with HDA9, HDA6, and BRAHMA (BRM)-containing SWITCH/SUC NONFERMENTING chromatin remodeling complex, association analyses are warranted to uncover the mechanisms underlying their roles in the plant HS response [82,83,84]. In tomato, HSFB1 recruits histone acetyltransferase 1 (HAC1) to chromatin, suggesting that the interaction of HSFB1 with HAC1 regulates gene expression in response to prolonged HS [85]. The histone chaperones AtASF1A (ANTI-SILENCING FUNCTION 1) and AtASF1B are involved in transcriptional activation in response to HS [86]. AtASF1A/B proteins are recruited onto chromatin, which was correlated with nucleosome removal and RNA polymerase II accumulation at the promoter and coding regions of *HSFs* and *HSPs* (Figure 4). Moreover, AtASF1A/B mediates the removal of H3K56ac marks from *HSR*s (heat stress response genes). In addition, the histone variant H2A.Z plays an important role in the thermosensory response in *Arabidopsis* [87]. Furthermore, the H2A.Z nucleosome regulates the binding of PIF4 (*PHYTOCHROME INTERACTING FACTOR 4*) to the FT (*FLOWERING LUCUS T*) promoter, thus mediating thermosensory activation of flowering [88]. PIF4 as a central regulator alters plant morphology (e.g., hypocotyl elongation, petiole elongation) at high temperatures by binding to the promoters of YUC8 (YUCCA8), TAA1 (TRYPTOPHAN AMINOTRANSFERASE OF ARABIDOPSIS), CYP79B2 (CYTOCHROME P450, FAMILY 79, SUBFAMILY B, POLYPEPTIDE 2), and SAUR (SMALL AUXIN UP RNA) 19–24 [63]. Therefore, H2A.Z may participate in regulation of plant morphology at high temperatures. However, whether H2A.Z modulates plant morphological acclimation to higher temperatures (e.g., extreme HS) and binds to other heat-response genes requires further investigation.

#### 3.3.3. Chromatin Remodeling

ATP-dependent chromatin remodeling complexes are involved in the plant response to HS [63]. The SWItch/sucrose non-fermentable (SWI/SNF) complex was first identified in *Arabidopsis*, and its key component *ARP6* (SWI complex) is essential for temperature sensing. Under heat and drought stress, overexpression of the SNF2/Brahma-type chromatin-remodeling gene *CHR12* (*CHROMATIN REMODELING*) caused growth arrest of flower buds and primary stems of *A*. *thaliana*, whereas *AtCHR12*-knockout *Arabidopsis* plants showed reduced growth arrest relative to the wild-type plants (Figure 4) [89]. This indicates that *CHR12* mediates temporary growth arrest of *Arabidopsis* under heat and drought stress. Additionally, upon resumption of a normal temperature, the H3-H4 chaperone CAF-1 (CHROMATIN ASSEMBLY FACTOR-1) participates in reloading of nucleosomes onto chromosomes in *A*. *thaliana* [76], indicating its importance in chromatin remodeling (Figure 4). In addition, HS relaxes the silencing of transposons by chromocenter decondensation mediated by HEAT-INTOLERANT 4 (HIT4), whereas most of these transposons are repressed rapidly by *MORPHEUS’ MOLECULE 1* (*MOM1*)- and *DECREASE IN DNA METHYLATION 1* (*DDM1*)-mediated chromatin remodeling [90].

#### 3.3.4. Epigenetic Memory

Epigenetic memory improves plant adaptation to various stress environments [61,91,92]. Histone modification and HSFA2 are important for HS memory in *A*. *thaliana*. The level of H3K4 methylation (H3K4me2/3), which is associated with transcriptional memory, was higher for at least 2 days after a priming heat shock [93]. Accumulation of H3K4 methylation is important for *HSR* expression and transcriptional HS memory, and this modification depends on HSFA2 (Figure 4). HSFA2 and H3K27me3 demethylase RELATIVE OF EARLY FLOWERING 6 (REF6) display a positive feedback loop to transmit long-term epigenetic memory in *A*. *thaliana* (Figure 4) [94]. In wheat, the level of lysine-specific histone demethylase 1 (LSD1) was upregulated in the progeny of heat-primed plants compared to that of non-heat primed plants, implicating histone modification in the induction of transgenerational thermo-tolerance by heat priming. HS-induced transgenerational epigenetic memory or phenotypic changes can be maintained for at least three generations [95,96]. In addition, the ONSEN retrotransposon, as mentioned above, is transcriptionally activated in plants exposed to HS. Interestingly, ONSEN transposition occurs more frequently in the progeny of RdDM mutants subjected to HS (Figure 4), indicating that RdDM-mediated epigenetic modification prevents transgenerational propagation of retrotransposons in plants [55,97].

## 4. Conclusions and Perspectives

Over the last decade, the mechanisms of the plant response to HS have been investigated in model plants. Our understanding of the regulatory networks involved in the plant HS response is mainly derived from model plants, such as *Arabidopsis*, rice, and tomato; few studies focused on non-model plants such as some agricultural crops and forestry trees (woody plants). Some genes may have experienced functional divergence during evolution, and homologous genes in different plants may have evolved different functions. Therefore, further studies of non-model plants are needed to enhance the understanding of the gene regulation networks underlying the plant HS response. Fortunately, advancements in genome sequencing, bioinformatics, and genetic transformation are enabling non-model plant research. By contrast, global warming is increasing the frequency of heat waves. Compared with short-term HS used in laboratory studies, the duration of warming events is longer, resulting in crop yield reductions. Therefore, efforts should focus on the plant response to long-term or prolonged HS, including transgenerational and multigenerational HS. This will facilitate evaluation of plant responses to climatic conditions and contribute to our ability to help plants cope with the warmer weather expected in the future.

The HS response mechanism at the transcriptional level of model plants has been gradually outlined. By contrast, the functions and interactions of important epigenetic regulatory factors in the plant HS response are unclear. Moreover, previous studies of epigenetic modification in response to HS focused on methylation and acetylation, whereas works on other epigenetic modifications—such as phosphorylation, ubiquitination, and SUMOylation (Small Ubiquitin-like Modifier, SUMO)—are scarce. Moreover, most methylation studies involved DNA, and little is known of RNA methylation in response to HS. Recently developed technologies, e.g., ATAC-seq (assay for transposase-accessible chromatin sequencing), histone modification, RNA modification (m6A/m1A/m5C), and single-cell RNAseq, will enhance research on epigenetic modifications under HS.

Another important question in the plant HS response is how heat is sensed. Identification of plant thermosensors may provide the missing link between the heat cue and the subsequent response [98]. Thermosensors must be activated directly by heat and require no upstream signaling components, excluding indirectly affected putative thermosensors. Heat affects DNA, RNA, protein, and lipids, and thus plant thermosensors may be composed of any one or a combination of these molecules. A prion-like domain in *Arabidopsis* rapidly shifts EARLY FLOWERING 3 (ELF3) between active and inactive states via phase transition [99]. Phase transition underlies the formation of biomolecular condensates in response to stimuli (e.g., temperature changes) [100]. Therefore, more research is needed to provide insight into the role of phase transition in the plant response to HS.
